# Mitochondrial Content, but Not Function, Is Altered With a Multimodal Resistance Training Protocol and Adequate Protein Intake in Leucine-Supplemented Pre/Frail Women

**DOI:** 10.3389/fnut.2020.619216

**Published:** 2021-01-22

**Authors:** Kathryn J. Jacob, Vita Sonjak, Sally Spendiff, Russell T. Hepple, Stéphanie Chevalier, Anna Perez, José A. Morais

**Affiliations:** ^1^Research Institute of the McGill University Health Center, Montreal, QC, Canada; ^2^Children's Hospital of Eastern Ontario Research Institute, University of Ottawa, Ottawa, ON, Canada; ^3^Department of Physical Therapy, Department of Physiology & Functional Genomics, University of Florida, Gainesville, FL, United States; ^4^Division of Geriatric Medicine, MUHC-Montreal General Hospital, McGill University, Montreal, QC, Canada; ^5^School of Human Nutrition, McGill University, Montreal, QC, Canada

**Keywords:** mitochondria, leucine, frailty, resistance training, muscle atrophy

## Abstract

**Background:** Frailty is a clinical condition associated with loss of muscle mass and strength (sarcopenia). Mitochondria are centrally implicated in frailty and sarcopenia. Leucine (Leu) can alter mitochondrial content in myocytes, while resistance training (RT) is the strongest stimulus to counteract sarcopenia and may enhance mitochondrial biogenesis.

**Objective:** We determined the effects of Leu supplementation and RT on mitochondrial content and function in pre/frail elderly women in a randomized double-blinded placebo-controlled study.

**Methods:** Nineteen pre/frail elderly women (77.5 ± 1.3 y, BMI: 25.1 ± 0.9 kg/m^2^), based on the Frailty Phenotype, underwent 3-months of RT 3×/week with protein-optimized diet and were randomized to 7.5 g/d of Leu supplementation or placebo alanine (Ala). Pre/post-intervention mitochondrial respiration, reactive oxygen species (ROS) production, calcium retention capacity (CRC), time to permeability transition pore (mPTP) opening, mitochondrial voltage-dependent anion channel (VDAC) protein content, leg press 1-repetition maximum (1RM), and 6-min walk test (6MWT) were measured.

**Results:** No time, supplementation, or interaction effects were observed for respiration, ROS, time to mPTP opening, and CRC. VDAC levels significantly increased in the Leu group post-intervention (*p* = 0.012). Both groups significantly increased leg press 1RM and 6MWT, with no effect of supplementation.

**Discussion:** Leu supplementation with 3 months of RT increased mitochondrial content. Future studies should investigate if there is an increase in mitochondrial turnover or a shift in quality control (mitophagy) in leucine supplemented pre/frail elderly women who undergo 12 weeks of RT.

**Clinical Trial Registration:** ClinicalTrials.gov, identifier: NCT01922167.

## Introduction

The World Health Organization has predicted that globally, by 2050 those over 60 years old will double from about 11 to 22% of the population. Numerically, this corresponds with ~2 billion people aged 60 or older, with 400 million of those being 80 years or older (1). Coinciding with this increase will be a surge in the occurrence of people with low strength and muscle mass (sarcopenia). If left unchecked, sarcopenia can further deteriorate to frailty and disability (2), cumulating in enormous economic burdens for healthcare systems on a global level (3). The gain in life expectancy of women is in part offset by increased frailty and years with disability (4).

A commonly-used clinical tool for diagnosing frailty is the Frailty Phenotype by Fried (5), which is comprised of five criteria: slowness, weakness, unintentional weight loss/sarcopenia, exhaustion, and sedentariness (5). Individuals meeting 1–2 criteria are deemed pre-frail, and those with three or more are frail. Sarcopenia is defined as muscular weakness and low muscle mass (atrophy) and is considered severe when muscle functioning is also compromised (e.g., slowness) (6). A decline in skeletal muscle function is also characteristic of aging and experimental evidence suggests that mitochondria are heavily implicated in the etiology of sarcopenia (7). A decline in *ex vivo* mitochondrial function (respiration and ROS production) with aging has been observed in our group (8), and another study mimicking physiological conditions has recently also observed a decline with aging (9). Damaged mitochondria not only have a hindered respiratory capacity but can also produce elevated ROS, resulting in further damage to mitochondria (10). They can also present with increased susceptibility to apoptosis *via* decreased CRC and/or mPTP opening, which may ultimately lead to fiber atrophy and hampered muscle performance (11, 12). Thus, mitochondria appear to be central to many detrimental age-related physiological changes. Indeed, both mitochondrial quality control (mitophagy and biogenesis), abundance, and function (13–16) have been shown to decline with aging. However, this is not always observed (14, 16–19) and similarly to respiratory capacity in our aforementioned study (8), many of these declines are thought to be attributed to lower mitochondrial abundance in older persons, likely attributed to increased sedentariness (as opposed to an inherent change with aging) (15, 20, 21). A recent study by our group demonstrated that mitochondria content was significantly lower in frail elderly women compared to young inactive controls (8). Furthermore, very little is known about mitochondrial functioning in aging women as opposed to men, though recent studies have suggested sex-dependent differences in mitochondrial physiology (22).

Leucine stimulates muscle protein synthesis by activating mammalian mechanistic target of rapamycin complex 1 (mTORC1) (23). mTORC1 signaling stimulates ATP-consuming processes (e.g., protein synthesis), necessitating mitochondrial function to replenish consumed ATP. Consequently, mTORC1 activity has been shown to correlate with mitochondrial activity (24). It has recently been shown that leucine has the capability to stimulate mitochondrial biogenesis, although the precise mechanisms remain unclear (25, 26). Additionally, in a recent study by Schnuck et al. (26) leucine treatment of cultured myoblasts resulted in heightened oxygen consumption which was attributed to a proportional increase in mitochondrial content. Therefore, leucine has received attention as a potential nutraceutical that may have benefits on mitochondrial function and/or content.

It is well-known that RT increases muscular mass and strength (27). RT has been shown to significantly increase mitochondrial content in elderly women (28) after 6 months of training. Furthermore, a recent study in elderly men and women showed that 8 weeks of RT significantly increased mitochondrial content in skeletal muscle (29). Finally, recent evidence in muscle of both healthy and pre/frail older adults at the transcriptome level has provided further insight regarding the plasticity of aging muscle to adapt to chronic resistance training (30).

Differences have been found in older persons who are active vs. sedentary [expending ≤1.5 METs while awake in a sitting, reclining, or lying posture (31)] in mitochondrial function (possibly due to reduced mitochondrial content with sedentariness) (32), although this is not always seen (21). Additionally, previous studies investigating the effects of RT on mitochondria in the elderly have not comprehensively assessed multiple aspects of mitochondrial function simultaneously (e.g., respiration, ROS production, mPTP sensitivity and mitochondria content) (28, 33, 34). To the best of our knowledge, it is currently unknown if mitochondrial function can be improved upon in a cohort of pre/frail elderly women who are sedentary at baseline and undergo a 12-week RT program with or without leucine supplementation. With the above context in mind, our research question was to determine the effects of leucine supplementation on mitochondrial function in pre/frail elderly women undergoing RT. We hypothesized that 12 weeks of RT would improve inherent mitochondrial function and/or increase the quantity of mitochondria, with an added benefit when supplemented with leucine compared to placebo. In addition, these improvements would occur in conjunction with functional improvements in skeletal muscle such as maximal strength and aerobic performance.

## Participants and Methods

### Study Design

The data presented in this article were obtained as a part of a larger study using the same subject group that participated in a registered randomized double-blinded placebo-controlled trial (ClinicalTrials.gov ID: NCT01922167). All participants underwent a 12-week multimodal high-intensity progressive resistance exercise training program and followed a protein-optimized diet (~1.2 g/kg/d). Half were randomized to receive leucine (2.5 g 3×/d) supplementation and the other half an isonitrogenous amount of alanine, an amino acid known not to stimulate muscle protein synthesis (1.7 g 3×/d) (35). All tests were performed before and after the intervention.

### Participant Recruitment and Screening

Frail or Pre-Frail community-dwelling elderly women (>65 y) according to the Fried Phenotype criteria (2001) were recruited from the Geriatrics outpatient clinic of the McGill University Health Center (MUHC) and advertisements posted in the local seniors' newspaper. As reported previously (36), 304 women were screened *via* telephone, 24 entered the study, and 19 completed the study. Of the five participants who left the study, two became ill with conditions unrelated to the study, one sustained an injury unrelated to the study, one moved out of province, and one was unable to maintain adherence to the protocol. The remaining 19 participants maintained adherence of at least 80% to both exercise program and supplement intake (36). Inclusion criteria consisted of non-disabled women who were cognitively intact with a Mini Mental State Examination score (MMSE) ≥24, body mass index (BMI) of 18.5–35 kg/m^2^, normal complete blood count, biochemistry, A1C, TSH, urine analysis, no diabetes determined by a 75-g oral glucose tolerance test (OGTT), negative serology for hepatitis and HIV, and normal chest X-ray and ECG results. Exclusion criteria were: dependence on walking aids, Geriatric Depression Score (GDS) short form <6 (37), substance abuse, eating disorders, active medical conditions other than skin cancer within 5 years, serum creatinine >110 μmol/L, hemoglobin (Hb) <110 g/L, and medications known to interfere with metabolic endpoint measurements (e.g., beta-blockers). The study was approved and monitored by the MUHC Human Research Ethics Board (REB code: 13–211-BMB). All participants read and signed an informed consent form before participation and screening visits. All outcome measurements were performed pre- and post- intervention.

### Intervention: Supplementation

Dietary protein intake was assessed using 3-d food diaries before and at the end of the study, with instructions to estimate portion sizes provided by a nutritionist and analyzed using the Food Processor SQL software (Version 10.11.0, ESHA Research, Salem OR). If needed, minor adjustments were made to their normal food intake to obtain a dietary protein intake of 1.2 g/kg/d. Participants were randomized into receiving either leucine or alanine supplementation by an independent source based on random generated numbers. Powdered supplements of leucine and isonitrogenous amounts of alanine were provided in sterile sealed screw-top 100 ml containers, of individual doses [2.5 g leucine (ProteinCo. QC, CA) and 1.7 g alanine (PureBulk® OR, USA)]. Participants were instructed to consume one complete dose of supplement at the onset of each main meal (breakfast, lunch, dinner) for the duration of the intervention. Log sheets were provided to track compliance and were collected every 2 weeks. Dietary protein intake was monitored *via* 24-h food recalls performed at least twice during the intervention.

### Intervention: Exercise Training

Participants trained three times per week on non-consecutive days, for ~1 h per session under supervision, as previously described (36). Briefly, participants warmed up walking on a treadmill for 10 min at a self-selected speed, followed by 5 min of range-of-motion and breathing exercises. Participants then performed resistance exercises targeting the major muscle groups of the upper and lower limbs: horizontal leg press, chest press, knee extension, and lateral pulldown. Participants performed 3 sets of 15 repetitions for each exercise and resistance was increased by 1–5 lbs (0.45–2.27 kg) when the participant could perform up to 15 repetitions with the proper technique. Training weights were determined to consistently be 60–80% of their 1-repetition maximum (1RM). Each session was ended with a 5-min, cool-down of stretching and breathing exercises.

### Physical Testing Outcome Measures

Physical testing was done at least 48 h before biopsy. Participants were instructed to refrain from vigorous physical activity 48 h prior to testing. Leg press 1RM was performed on a horizontal leg press (C-403, Atlantis Inc. Laval, QC) by trained kinesiologists according to a standard protocol (38). The 6-min walk test (6MWT) was performed over a 30-m hallway (60-m course) according to a standard protocol (39).

### Mitochondrial Function Outcome Measures

Methodology outlined below has been previously published (8, 21).

#### Sample Collection

Participants presented to the clinical unit after an overnight fast, and at least 48 h after the last bout of physical activity. Skeletal muscle samples were obtained from the lateral portion of the *vastus lateralis* ~20 cm above the knee, 4–5 cm apart using the Bergström needle biopsy technique (40). After fat removal, samples were separated into aliquots. Due to limited muscle tissue from a few participants, some measurements were only performed on a subset of muscle biopsy specimens. The sample size used for each measurement is indicated in the results section of each technique used in this study.

#### Preparation of Saponin-Permeabilized Muscle Fiber Bundles

About 70–100 mg of fresh muscle tissue was put in ice-cold buffer A [2.77 mM CaK_2_EGTA, 7.23 mM K_2_EGTA, 6.56 mM MgCl_2_, 0.5 mM dithiothreitol (DTT)], 50 mM KMES, 20 mM imidazol, 20 mM taurine, 5.3 mM Na_2_ATP, 15 mM phosphocreatine, pH 7.3). Manual dissection under a stereomicroscope served to isolate 2–6 mg fiber bundles that were subsequently chemically permeabilized for 30 min in buffer A with 50 μg ml^−1^ of saponin at 4°C with gentle agitation.

#### High Resolution Respirometry

Saponin-permeabilized fiber bundles were washed 3 × 10 min in buffer B (2.77 mM CaK_2_EGTA, 7.23 mM K_2_EGTA, 1.38 mM MgCl_2_, 3 mM K_2_HPO_4_, 0.5 mM DTT, 20 mM imidazole, 100 mM KMES, 20 mM taurine and 2 mg/mL BSA, pH 7.3). Respiration of 3–5 mg of wet weight fibers was then measured at 37°C with continuous stirring in 2 mL of buffer B in an Oxygraph-2 K (Oroboros, Innsbruck, Austria), under hyperoxygenated conditions to prevent oxygen diffusion limitation. Respiration was recorded after addition of the following substrates: glutamate (10 mM) + malate (5 mM) (state 2), ADP (2 mM) (maximal state 3 respiration driven by complex I substrates), succinate (10 mM) (state 3 respiration driven by complex I and II substrates), Cytochrome c (5 mM) (assess mitochondrial outer membrane integrity), antimycin A (10 μM) (inhibit complex III). The acceptor control ratio (ACR) was calculated as respiration driven by the addition of ADP (state 3) divided by respiration driven by glutamate and malate (state 2) as an indication of mitochondrial coupling.

#### Mitochondrial ROS Emission

Permeabilized fiber bundles for ROS emission were washed 3 × 10 min in buffer Z (110 mM KMES, 35 mM KCl, 1 mM EGTA, 3 mM MgCl_2_, 10 mM K_2_HPO_4_ and 0.5 mg/mL BSA, pH 7.3). ROS emission was determined by recording the rate of generation of the fluorescent compound resorufin (product of the oxidation of Amplex red by H_2_O_2_ and fatty acid hydroperoxides released from the mitochondria). Fluorescence was measured using an F-2500 fluorescence spectrophotometer (Hitachi, Tokyo, Japan) at an excitation/emission wavelength of 563/587 nm. Following baseline measurements, permeabilized fiber bundles (3–6 mg) were added to 600 μl of solution Z, with 5.5 μM Amplex red and 1 U per mL horseradish peroxidase (HRP) in a magnetically stirred quartz cuvette at 37°C. The substrates were then added sequentially: glutamate (10 mM) + malate (2 mM), succinate (10 mM), ADP (10 μM), ADP (100 μM) and Antimycin A (10 μM). ROS emission was determined using a standard curve constructed on the same day of the experiment using known concentrations of H_2_O_2_. Fiber bundles were retrieved and stored at −80°C until western blot analysis.

#### Time to mPTP Opening and CRC

Permeabilized fiber bundles for mPTP function were washed 3 × 10 min in solution C (80 mM KMES, 50 mM Hepes, 20 mM taurine, 0.5 mM DTT, 10 mM MgCl_2_ and 10 mM ATP, pH 7.3) and then had myosin extracted using solution D (800 mM KCl, 50 mM Hepes, 20 mM taurine, 0.5 mM DTT, 10 mM MgCl_2_ and 10 mM ATP, pH 7.3) without agitation. Myofibers were then washed 3 × 10 min in CRC solution (250 mM sucrose, 5 μM EGTA-Tris Base and 10 mM Tris-MOPS, pH 7.4). The bundles (4–6 mg) were then added to 600 μL of CRC solution containing glutamate (5 mM), malate (2.5 mM), phosphate (10 mM), oligomycin (0.5 nm) and Calcium Green^TM^-5 N, Hexapotassium Salt (0.001 mM) (Life Technologies, USA). The Ca^2+^ uptake by the mitochondria was measured by monitoring the decrease in fluorescence observed corresponding to reduction in free Ca^2+^ in the solution when Ca^2+^ was taken up into the mitochondria. The time point at which the fluorescence began to increase was taken as the time to pore opening, while the amount of Ca^2+^ up taken by the mitochondria prior to pore opening as the CRC. Fluorescence was detected using an F-2500 fluorescence spectrophotometer (Hitachi, Tokyo, Japan) at an excitation/emission wavelength of 505/535 nm. The Ca^2+^ concentration in the solution was determined using a calibration curve of known Ca^2+^ concentrations performed on the day of the experiment. Fiber bundles were retrieved and stored at −80°C until western blot analysis.

Analysis of all mitochondrial function experiments was performed using bespoke software created in-house using Igor Pro Software (Wavemetrics, https://www.wavemetrics.com) (14).

#### Mitochondrial Protein Content

Western blotting for voltage-dependent anion channel (VDAC, an outer membrane protein) was used as a marker of mitochondrial content in all retrieved fiber bundles. Five to 36 mg of muscle tissue was homogenized 2 × 45 s with a robot homogenizer (Minibead-beater, Biospec Products, USA) with 1.4 mm ceramic beads and a 10× volume of RIPA extraction buffer [50 mM Tris base, 150 mM NaCl, 1% Triton X-100, 0.5% sodium deoxycolate, 0.1% sodium dodecyl sulfate, and protease inhibitor cocktail tablet (1 tablet/10 μl of RIPA buffer, ROCHE)]. Samples were then incubated at 4°C for 2 h and then centrifuged at 12,000*g* for 20 min at 4°C. The supernatant was collected and protein content was assessed *via* Bradford assay. Immunoblotting was performed with 15 μg of tissue protein diluted in 4× Laemli buffer and extraction buffer and boiled at 95°C for 5 min. 24 μl of sample was then loaded onto a 12% acrylamide gel, electrophoresed by SDS-PAGE and transferred at 4°C to polyvinylidene fluoride membranes (Amersham Hybond ECL, GE Healthcare Life Sciences). The membranes were blocked in 5% semi-skimmed milk for 1 h at room temperature and incubated over night at 4°C with a primary mouse monoclonal anti-VDAC antibody (ab14734, dilution 1:1,000, Abcam) diluted in 5% BSA. Total protein loading (Mini-PROTEAN® TGX Stain-Free^TM^ Precast Gels, Bio-Rad Laboratories, Inc. PA, USA) was used to normalize protein loading (8, 41). Membranes were incubated with rabbit anti-mouse HRP-conjugated secondary antibody diluted in 5% milk (ab6728, diluted 1:2,000, Abcam) for 1 h at room temperature. Protein bands were detected with SuperSignal^TM^ West Pico Chemiluminescent Substrate (Thermo Scientific, Waltham, MA, USA) and imaged with a BIO-RAD image system (Bio-Rad ChemiDoc^TM^MP Imaging System). Identification and quantification of protein bands was performed using Image Lab^TM^ Software, Version 6.0.1 (Bio-Rad Laboratories, Inc. PA, USA), where both bands at the approximate molecular mass of VDAC in a given blot were combined to obtain an index of the quantity of VDAC protein (41).

All mitochondrial function values obtained in the study were expressed relative to VDAC to normalize the respiratory capacity per mitochondrion (i.e., the intrinsic organelle function), except time to mPTP opening which is independent of mitochondrial content (42). VDAC protein content was chosen to normalize mitochondrial function data over mitochondrial enzyme function because age *per se* (43, 44) and changes in physical activity can alter enzymatic function (45–47), which may not accurately represent changes in mitochondrial content (47).

### Statistical Analysis

Normality was determined using the Shapiro-Wilk test. Independent *t*-tests were used to determine differences between the two groups at baseline. Two-factor repeated measures ANOVA was used to determine the leucine supplementation (group) and exercise training (time) effects. When significant interaction effects were observed, *post-hoc* comparisons were performed using the Sidak test. Significance was set at *p* ≤ 0.05. Statistical analyses were performed using Prism 7.0a (GraphPad Software, Inc. CA, USA).

## Results

Study participant profiles have been published elsewhere (36). Respectively, participants did not differ at baseline for any characteristics (Table 1).

**Table 1:** Clinical characteristics of pre/frail women by supplement group at baseline.

**Characteristics**	**Ala**	**Leu**
*n*	9	10
Age (y)	76.2 ± 1.8	78.7 ± 2.1
Weight (kg)	61.8 ± 2.5	62.9 ± 2.9
BMI (kg/m^2^)	23.8 ± 1.0	26.2 ± 1.3
Number of frailty criteria	2.7 ± 0.3	2.6 ± 0.3
% Fat	36.0 ± 2.2	41.3 ± 1.5
LBM (kg)	38.1 ± 1.3	35.2 ± 1.4

*Values presented as mean ± SEM, BMI, body mass index; LBM, lean body mass; % Fat, percent body fat*.

### Diet

As reported previously (36), no group, time, or interaction effects were observed for dietary protein intake. Similarly, no group, time, or interaction effects were observed for dietary leucine intake (excluding consumed supplement) pre- and post-intervention (Ala: 5.8 ± 0.2 vs. 5.5 ± 0.3 g, Leu: 5.1 ± 0.3 vs. 5.4 ± 0.4 g, pre vs. post, respectively).

### Leg Press and 6MWT

**Figure 1:**
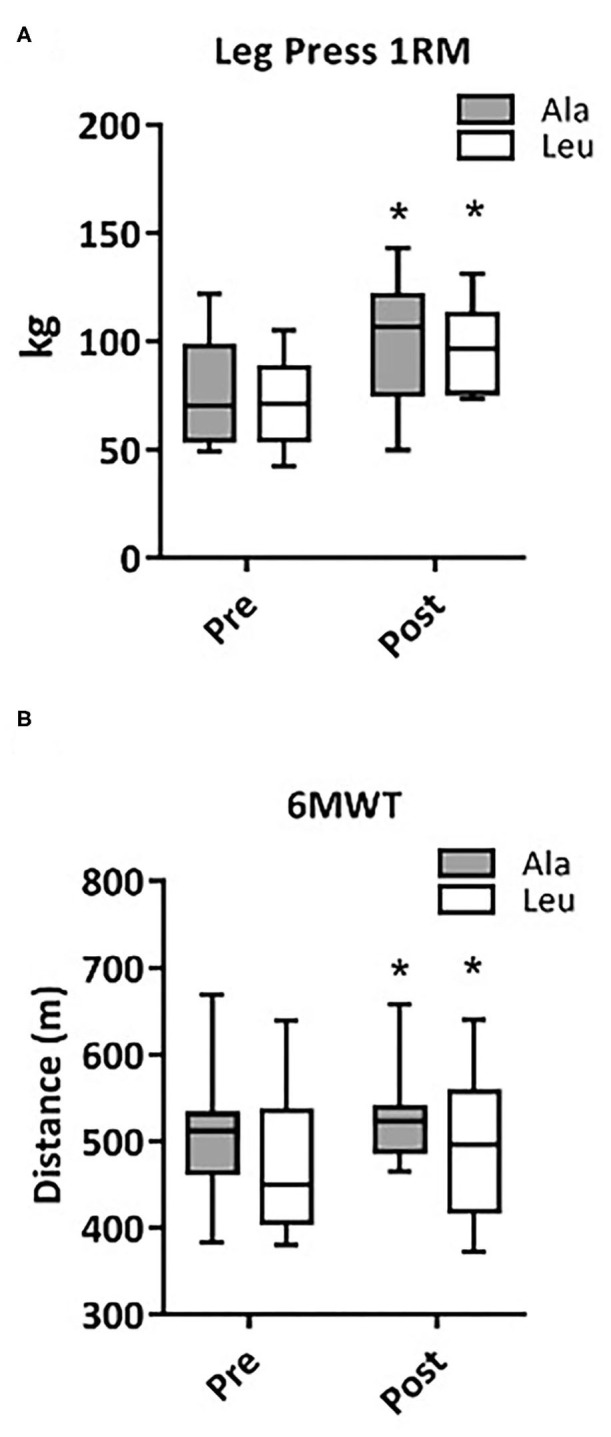
Changes in maximal anaerobic muscular function (Leg Press 1RM, Ala: *n* = 9, Leu: *n* = 10) **(A)** and muscular aerobic function (6MWT, Ala: *n* = 8, Leu: *n* = 10) **(B)** in pre/frail women with and without leucine supplementation before and after 12 weeks of resistance exercise training. *denotes interaction effect, *p* < 0.05. Ala, alanine supplemented group (control), Leu, leucine supplemented group; 1RM, 1 repetition maximum; 6MWT, 6-meter walk test.

Leg Press 1RM as a surrogate measure of Mmaximal anaerobic muscular function, significantly increased from pre to post intervention (time effect, p < 0.0001), with no group or interaction effect (Ala: 76.0 ± 8.1 vs. 99.6 ± 9.2 kg, Leu: 71.3 ± 5.9 vs. 96.4 ± 5.9 kg, pre vs. post, respectively, Figure 1A). The distance walked during the 6MWT as a surrogate measure for Mmuscular aerobic function significantly increased from pre to post intervention (time effect, *p* = 0.039), with no group or interaction effect (Ala: 508 ± 28.8 vs. 538.0 ± 20.1 m, Leu: 474.4 ± 27.3 vs. 497.0 ± 26.6 m, respectively, Figure 1B).

### VDAC

**Figure 2:**
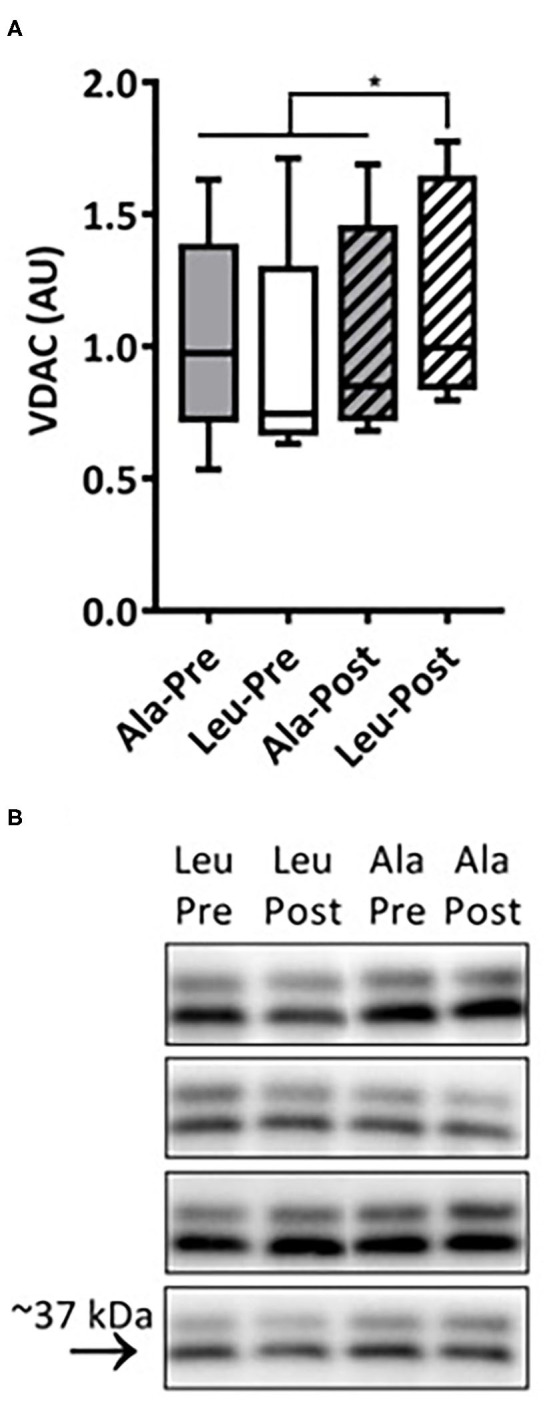
**(A)** VDAC protein expression determined by Western Blot in pre/frail women with and without leucine supplementation before and after 12 weeks of resistance exercise training. Ala: *n* = 8, Leu: *n* = 7. No significant group or time effects were observed. *denotes interaction effect, *p* < 0.05. **(B)** Representative Western Blot, fourth column is ladder. Ala, alanine supplemented group (control); Leu, leucine supplemented group.

The VDAC protein expression, a marker for mitochondrial content (Figure 2A) did not differ pre-training and increased only in the Leu group (*n* = 7) with exercise training (interaction *p* = 0.034, time effect, *p* = 0.020). Ala (*n* = 8) remained unchanged from baseline.

### Respiration

**Figure 3:**
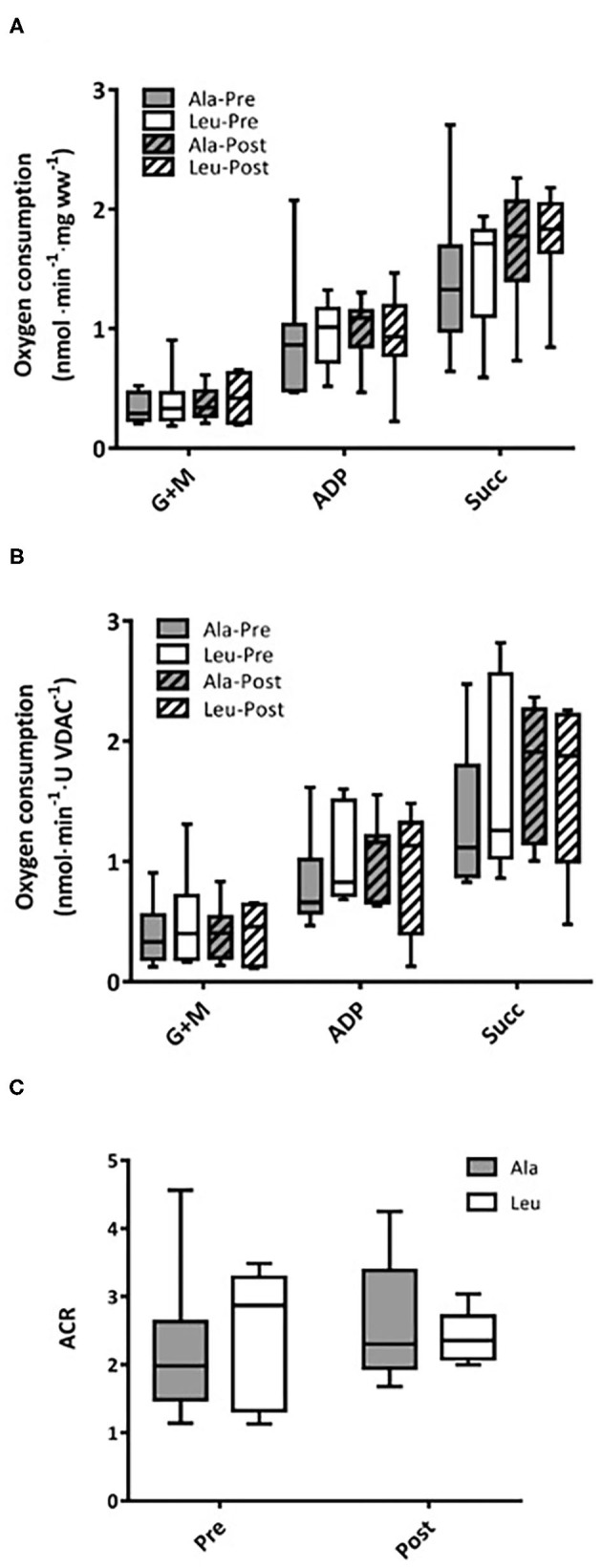
Mitochondrial respiration in permeabilized fiber bundles of *vastus lateralis* normalized to **(A)** fiber wet weight, and **(B)** VDAC abundance as a marker of mitochondrial content in pre/frail women with and without leucine supplementation before and after 12 weeks of resistance exercise training. **(C)** Acceptor control ratio obtained by the division of ADP by Glutamate+malate respiration. (A) Ala: *n* = 8, Leu: *n* = 7, **(B)** Ala: *n* = 8, Leu: *n* = 6, **(C)** Ala: *n* = 8, Leu: *n* = 5. No significant group, time, or interaction effects in respiration in any state were observed. ADP, adenosine triphosphate; Ala, alanine supplemented group (control); G + M, glutamate + malate; Leu, leucine supplemented group; Succ, succinate; ACR, acceptor control ratio.

Respiratory capacity did not change over time or differ between groups, and no interaction effects were observed for any substrate, normalized to bundle wet weight (Ala: *n* = 8, Leu *n* = 7) (Figure 3A) or mitochondrial content (VDAC, Ala: *n* = 8, Leu *n* = 6) (Figure 3B). ACR did not differ over time or between groups, and no interaction effects were observed (Ala: *n* = 8, Leu *n* = 5) (Figure 3C).

### ROS

**Figure 4:**
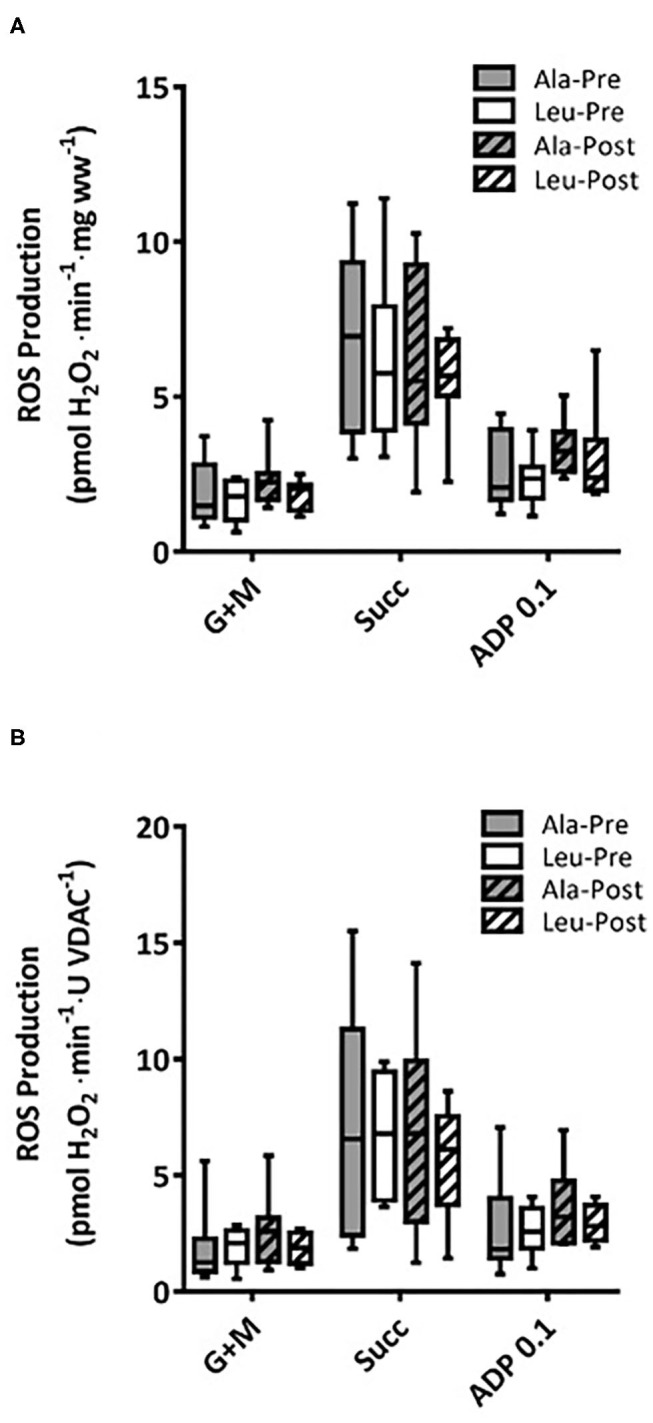
Mitochondrial ROS production in permeabilized fiber bundles of vastus lateralis normalized to **(A)** fiber wet weight, and **(B)** VDAC abundance as a marker of mitochondrial content in pre/frail women with and without leucine supplementation before and after 12 weeks of resistance exercise training. **(A)** Ala: *n* = 8, Leu: *n* = 7, **(B)** Ala: *n* = 7, Leu: *n* = 6. No significant group, time, or interaction effects in ROS production in any state were observed. ADP, adenosine triphosphate; Ala, alanine supplemented group (control); G + M, glutamate + malate; Leu, leucine supplemented group; Succ, succinate.

ROS production did not differ over time or between groups, and no interaction effects were observed for any substrate, normalized to bundle wet weight (Ala: *n* = 8, Leu *n* = 7) (Figure 4A) or mitochondrial content (VDAC, Ala: *n* = 7, Leu *n* = 6) (Figure 4B).

### CRC and mPTP

**Figure 5:**
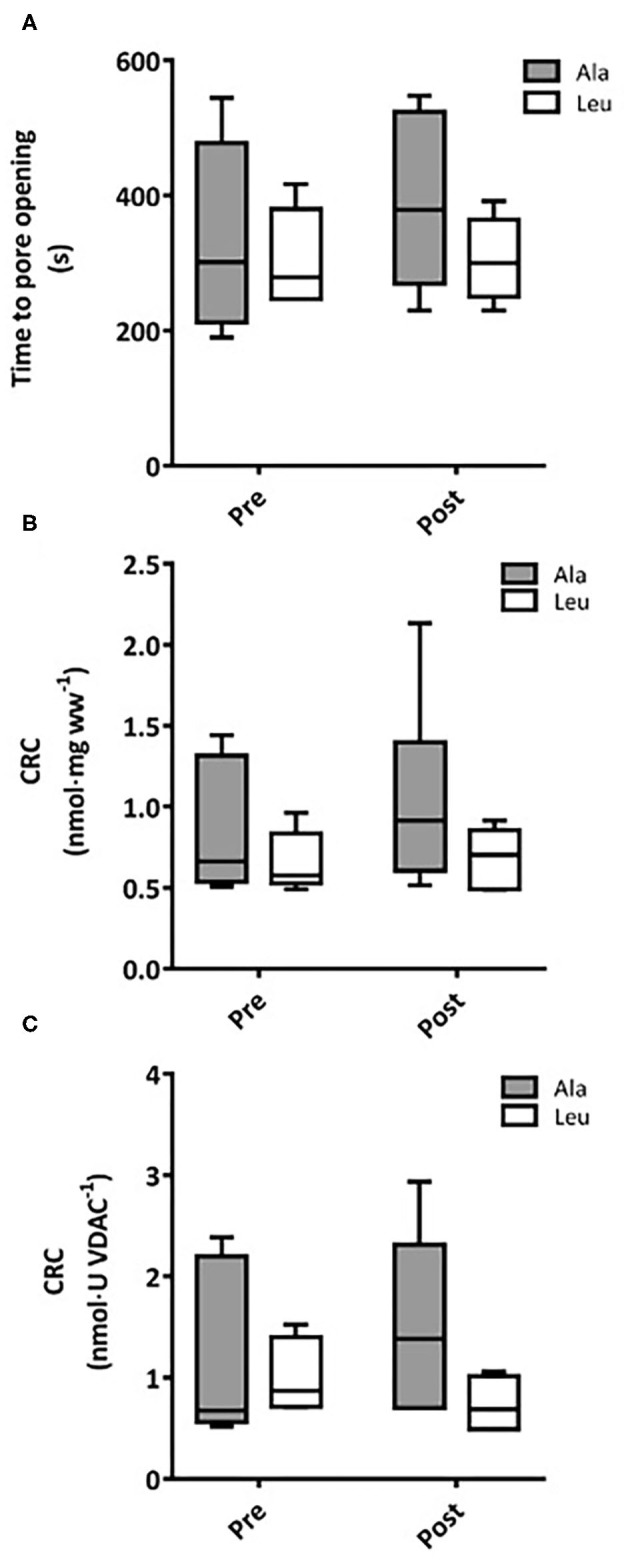
**(A)** Time to mitochondrial permeability transition pore (mPTP) opening in permeabilized fiber bundles of vastus lateralis muscle. Calcium retention capacity (CRC) in permeabilized fiber bundles of vastus lateralis normalized to **(B)** fiber wet weight, and **(C)** VDAC abundance as a marker of mitochondrial content in pre/frail women with and without leucine supplementation before and after 12 weeks of resistance exercise training. **(A)** Ala: *n* = 5, Leu: *n* = 6, **(B)** Ala: *n* = 6, Leu: *n* = 5, **(C)** Ala: *n* = 5, Leu: *n* = 4. No significant group, time, or interaction effects in time mPTP opening or CRC were observed. Ala, alanine supplemented group (control); Leu, leucine supplemented group; mPTP, mitochondrial permeability transition pore; CRC, calcium retention capacity.

Time to pore opening did not differ over time or between groups, and no interaction effects were observed (Ala: *n* = 6, Leu *n* = 5) (Figure 5A). CRC did not differ over time or between groups, and no interaction effects were observed normalized to bundle wet weight (Ala: *n* = 6, Leu *n* = 5) (Figure 5B) or mitochondrial content (VDAC, Ala: *n* = 5, Leu *n* = 4) (Figure 5C).

## Discussion

It is currently unknown if mitochondrial functioning can be improved by resistance training in combination with leucine supplementation in women experiencing the clinical entity of frailty or pre-frailty. We measured several states of substrate-supported mitochondrial respiration and ROS production, as well as CRC and time to mPTP opening as indices of sensitivity to undergo mitochondrial permeability transition, an event associated with release of proteins that cause death in mononucleated cells in frail and pre-frail elderly women who consumed adequate dietary protein and underwent 12-weeks of RT with or without leucine supplementation. We included two indices of muscular function, aerobic (6MWT) and anaerobic (Leg Press 1RM), measured pre and post intervention. Our main findings were that 12 weeks of RT: (1) had no effect on mitochondrial respiration capacity, ROS production, or CRC regardless of leucine supplementation, (2) significantly increased VDAC protein levels in Leu supplement group, and (3) significantly increased both Leg Press 1RM and 6MWT, with no added effect of leucine supplementation.

In our recent study we showed that VDAC was reduced in vastus lateralis muscle of a similar cohort of pre-frail and frail elderly women compared with young counterparts, suggesting reduced mitochondrial content (8). We observed an increase in mitochondrial content in the Leu, and not Ala, group post-intervention as evidenced by VDAC protein expression. Although VDAC's use as a marker of mitochondrial content has been established in aging (8, 21), we do realize that a single mitochondrial protein may not truly represent the dynamics of hundreds other proteins that comprise the mitochondrion and could be affected by RT and/or amino acid supplementation. The gold standard to assess mitochondrial content is mitochondrial volume density which is measured by electron microscopy. The only study that used this measure in older women (70y of age) found only a trend in the reduction of mitochondrial volume density in moderately physically active older women compared to activity matched young controls (48). Nevertheless, increased VDAC in the leucine group but not in the alanine placebo group may suggest an increased level of mitochondrial biogenesis and/or suppression of mitophagy. Therefore, we observed an inconsistent effect of exercise training between the supplement groups. Although RT has been associated with increased mitochondrial biogenesis in young healthy men (20), elderly men and women (29), and postmenopausal women (28), it is interesting that in the current study the placebo (Ala) supplementation group did not increase mitochondrial content following exercise. It is unknown if alanine has inhibitory effects on exercise-stimulated mitochondrial biogenesis. We are aware of only one study alluding to this question in 20-month-old rats that underwent 8 weeks of voluntary wheel running with or without HMB (beta-hydroxy-beta-methylbutyrate, a leucine metabolite) plus alanine supplementation (49). Unexpectedly, in that prior study the supplemented group had reduced amounts of PGC-1α in the medial gastrocnemius as well as reduced markers of autophagy in comparison to the HMB group post-intervention. The mechanism underlying these results was not determined in that study. Leucine has been previously shown to stimulate PGC-1α expression *via* activation of SIRT1's action on AMPK, resulting in mitochondrial biogenesis in cultured myotubes (25, 50, 51). However, this increase in mitochondrial content has not been previously demonstrated in human muscle tissue *ex vivo*. Leucine can also increase peroxisome proliferator-activated receptor alpha (PPARα) and PPARβ/δ expression, leading to PPARβ/δ-dependent increases in mitochondrial content and oxygen consumption in cultured myotubes (26). Another potential explanation is that in pre/frail elderly women, RT alone is not able to increase mitochondrial content, which is in agreement with a recent study where VDAC content did not change in older men who underwent 12 weeks of RT (9). Thus, to the best of our knowledge, this is the first study demonstrating that leucine supplementation in conjunction with RT increases mitochondrial content (possibly *via* either increased biogenesis and/or reduced mitophagy) in elderly women. Future studies should also investigate how leucine supplementation impacts the mitochondrial biogenesis response with exercise interventions in frail vs. sarcopenic vs. healthy-elderly persons to determine if the observed effect is unique to frailty.

We observed no changes in mitochondrial respiration with the intervention when normalized to wet-weight or VDAC (mitochondrial content), although as mentioned earlier we noticed greater mitochondrial content in the leucine group after RT. Interestingly, this did not result in improved mitochondrial respiration suggesting that the new mitochondria may not be functioning well-enough on an individual basis to improve overall respiration. A potential factor contributing to the latter notion may be a combination of low sample size and interindividual heterogeneity in response to RT (52). However, the intensity of the training was sufficient to improve muscle performance based upon increases in 1RM and 6MWT, with no difference between supplement groups. Accounting for mitochondrial quantity, it has been demonstrated that the respiratory capacity of skeletal muscle appears to be preserved with aging in healthy active older men (14), while a mild impairment in mitochondrial respiratory capacity was observed in older inactive men (21). Furthermore, mitochondrial respiration (state 4) normalized per wet weight (and not mitochondrial content) was reduced in old vs. young persons, and further reduced in low vs. highly functioning elderly persons (13). Importantly, these previous studies have investigated either men only or combined groups of sexes, while women remained understudied. Our group is the first to report mitochondrial respiration in an all-female cohort. We have recently shown that when normalized to wet weight, state 3 driven respiratory capacity was reduced in inactive pre-frail and frail elderly women (a cohort comparable to the current study participants at pre-intervention) when compared to younger controls (8), but these differences disappeared when normalized to mitochondria content. A recent study (19) showed no differences in mitochondrial respiration across a wide range of ages and cardiorespiratory fitness when normalized for mitochondrial content. A subsequent study by the same group concluded that mitochondrial respiration is affected more by chronic physical activity status rather than chronological age (16). The nature of the chronic physical activity in their study, being aerobic, could account for the greater mitochondrial content in their active participants, and thus account for superior mitochondrial respiration. Our results are in agreement with these and other groups which have attributed any decline in mitochondrial respiration to mitochondrial quantity, rather than an intrinsic impairment of mitochondrial respiratory functioning (14, 20), although this is not a uniform finding (21). It was recently shown using a novel methodology, that although maximal levels of respiration were not different between young and older men with similar VO_2peaks_, reduced respiration was seen in older men compared to young over a range of biologically relevant ADP levels suggesting a reduced sensitivity to ADP with aging (9). Furthermore, 12 weeks of RT increased both maximal (state 3) mitochondrial respiration (normalized to wet weight) as well as respiration at submaximal ADP concentrations (9). The aforementioned study observed an improvement in maximal respiration per unit of muscle following RT, and thus, perhaps we did not see improvements in the current study because maximal capacity of mitochondrial respiration was measured with saturating levels of ADP, and not assessed under physiological levels of ADP as done in this recent study.

Mitochondria are a significant source of ROS production within cells and therefore an impairment in their metabolism has been suggested to play a role in the development of frailty. Indeed, it has been shown that skeletal muscles of elderly persons with reduced physical function are more susceptible to oxidative damage (13). However, there is great discrepancy in the literature about whether or not ROS production increases with aging (14, 17, 53). It has recently been shown that ROS emission in *vastus lateralis* muscle is markedly higher in pre-frail and frail compared to young inactive women after normalization to mitochondrial content (8). If ROS emission is greatest in sedentary muscle, then increased muscular contraction could reduce ROS emission (10). However, we observed no changes in mitochondrial ROS production after 3 months of RT when normalized to wet weight or mitochondrial content in any of the supplemental groups in our pre-frail and frail women. This is consistent with findings of a recent study in healthy older men (≥60 y) who underwent a 12-week RT program (without supplementation) showing no changes in ROS production post-intervention (33). In addition, ours is the first study performed in pre-frail and frail older women to show that ROS emission was not ameliorated with leucine supplementation. To the best of our knowledge, only one study has investigated if leucine impacts ROS emission. It has been found that 5 days of a leucine-rich diet resulted in decreased ROS production in both *in vivo* and cultured epithelial cells of piglets (54). This was attributed to leucine causing a metabolic shift from oxidative phosphorylation to glycolysis by activation of the mTOR-HIF-1α pathway. However, no changes in ROS production were seen in the current study, possibly due to the different tissues, ages, and species studied. The aforementioned study in older men who underwent 12 weeks of RT (9) yielded results differing from our own. Following 12 weeks of RT maximal ROS production increased at saturating levels of ADP, while ROS emission was attenuated at submaximal levels of ADP. Thus, our results highlight a possible difference between sexes, as to our knowledge no studies exist investigating sexual dimorphism and ROS production in saponin-permeabilized myofibers with aging.

Leucine can directly activate SIRT1 which subsequently phosphorylates AMPK, and downstream of that, PGC-1α (a major modulator of mitochondrial biogenesis) and SIRT3 (25). SIRT1 has been shown to deacetylate Mfn2 resulting in increased mitophagy (55). SIRT3 can deacetylate a component of the mPTP resulting in the inhibition of mPTP-mediated apoptosis and increased mitophagy in cardiomyocytes (56). However, in a recent study in obese adolescent males, the authors determined that aerobic, and not resistance training, is necessary to induce SIRT3 in skeletal muscle (57). To our knowledge this is the first study to investigate the impact of any type of exercise training on CRC and mPTP opening. It remains to be determined if endurance training would have a different effect on mPTP sensitivity with or without leucine supplementation. Future studies should investigate this unanswered question, as well as including measurements of markers of mitochondrial quality control (mitophagy, fusion/fission) and PGC-1α.

A potential limitation of the current study is that it has recently been shown that high variability can exist in mitochondria respiration measurements, and thus detecting changes in mitochondria respiration may require larger sample sizes (58). The nature of the population investigated (pre/frail, low muscle mass, etc.) and the difficulty recruiting and retaining such valuable participants remains a challenge for this field of research. Another potential limitation of the current study is that changes in fiber type were not considered. A fiber-type profile with predominantly slow-twitch muscle would be more oxidative, while predominantly fast-twitch fibers could produce more ROS (12, 42). Additionally, depending on the type of physical activity, that is, endurance vs. resistance, there are divergent effects on the different fiber types (12). A prominent future direction to the current study is to examine the effects of our intervention on muscle denervation. Our group has recently shown that inactive pre-frail and frail elderly women have a greater intrinsic mitochondrial ROS emission compared to young inactive women, possibly due to an increase in denervation-induced mitochondrial ROS production (8). It remains to be determined if an exercise intervention such as that of the current study would have the capacity to improve myofiber reinnervation as well as ameliorate denervation-induced mitochondrial ROS production.

In conclusion, 12-weeks of RT in pre/frail elderly women with and without leucine supplementation increased leg strength and walking distance, while mitochondrial content was increased with RT only in combination with leucine supplementation. A more comprehensive understanding of the functioning of the mitochondria for different sexes, ages, and disease states is critical for mitochondria to be a viable therapeutic target for age-related conditions including sarcopenia and the development of frailty, therefore contributing the health span.

## Data Availability Statement

The raw data supporting the conclusions of this article will be made available by the authors, without undue reservation.

## Ethics Statement

The studies involving human participants were reviewed and approved by McGill University Health Center Human Research Ethics Board (Study REB code: 13-211-BMB). The patients/participants provided their written informed consent to participate in this study.

## Author Contributions

KJ, JM, and RH designed the study. KJ recruited and screed participants and was responsible for exercise testing as well as exercise and dietary intervention, performed statistical analysis and data interpretation with guidance from JM, SC, RH, and VS, and wrote the manuscript and was revised by SC, SS, RH, and JM. KJ, VS, AP, and SS performed mitochondrial function measurements. All authors contributed to the article and approved the submitted version.

## Conflict of Interest

The authors declare that the research was conducted in the absence of any commercial or financial relationships that could be construed as a potential conflict of interest.
